# Editorial: Cardiovascular Anthropometry For Large Scale Population Studies Volume II

**DOI:** 10.3389/fcvm.2026.1853399

**Published:** 2026-05-28

**Authors:** Basil N. Okeahialam, Okechukwu S. Ogah

**Affiliations:** 1 Department of Medicine, Jos University Teaching Hospital, Jos, Nigeria; 2 Department of Medicine, University College Hospital, Ibadan, Nigeria

**Keywords:** anthropometry, atherogenic index of plasma, body roundedness index, cardiovascular diseases, triglyceride glucose index, weight adjusted weight index

Obesity and overweight have long been known to be harbingers of cardiometabolic diseases. The body mass index (BMI) has stood the test of time as a measure of obesity/overweight. With time however, it became obvious that it could wrongfully classify one at risk of cardiometabolic disease, because it factored in all weight; whereas it is the fat component that is critical. More relevant is the fact that not all fat components but fat in ectopic sites that matter. This gave rise to a flurry of research seeking to identify more critical anthropometric measures as well as what worked more for different populations with different phenotypes. Given the relevance of cardiovascular anthropometry in cardiometaboilc disease prediction, interests have continued to be generated in this subject that strongly resonates in preventive cardiology (1).

This issue of Frontiers in Cardiovascular Medicine is the second dedicated to articles in cardiovascular anthropometry (traditional and non-traditional) as well as non-physical biomarkers of cardiovascular diseases. This came about because the first issue was adjudged successful. The contributions are as varied as they are exciting.

One such non-traditional anthropometric index is Body Roundedness Index (BRI). In their work, Wang et al. related it to mortality and life expectancy in a metabolic syndrome population, and found that it predicted all-cause mortality comparably with BMI. Its association with all-cause and cardiovascular mortality demonstrated a “U” shape pattern. As posited by Jin (2), BRI more poignantly reflects body shape and fat distribution; aspects more closely related to cardiometabolic disease rates and prognosis.

Atherogenic index of plasma (AIP) is a biomarker of atherosclerotic cardiovascular disease (ASCVD). Its relationship with hypertension was explored using BMI to see if there was any mediating role. According to Zhang et al. AIP is an independent predictor of hypertension in adults working through BMI. Yu et al. also studied this metric by interrogating the association between it and BMI in cardiometabolic diseases among middle aged and aged Chinese; finding that both AIP and BMI increase the risk of cardiometabolic diseases with BMI as mediator. This biomarker as shown by Zhang et al. goes beyond establishing a direct link with cardiovascular diseases into the realm of Cardiovascular—Kidney—Metabolic Syndrome, a new entity which shows the renal consequences of ASCVD.

Triglyceride Glucose Index (TGI) is a biomarker of cardiovascular diseases, deriving from insulin resistance (IR). According to Bao et al, it predicts dynamic progression through ASCVD to cardiovascular morbidity. The ability of TGI to predict cardiovascular diseases has been highlighted by Okeahialam and Sirisena (3) where a critical cut off value was determined above patients’ propensity to developing ASCVD rises.

Liu et al. studied association of anthropometric indicators of hypertension with subclinical cardiovascular diseases among obese adolescents. Using carotid-intima-media thickness (CIMT) to mirror sub-clinical ASCVD, they found significant association between CIMT and anthropometric measures in obese adolescents. They then recommended early intervention even at that age range. Okeahialam et al. (4) had shown that such vascular markers as CIMT could suggest risk of ASCVD and when skill and equipment are available, CIMT could be used to determine when to initiate preventive measures.

Another novel anthropmetric index, weight adjusted waist index was derived by Pimenta et al. and used to study patients with cardiovascular diseases. It is said to overcome the limitations of traditional anthropometric indices. It combines waist circumference (WC) and body weight; and clearly suggested a relationship with cardiovascular diseases, hence it being recommended as an alternative index. Sadafi et al. (5) have shown that it out-performs the traditional anthropometric indices BMI and WC separately and should have less chances of misclassifying individuals in need of intervention. Zhang et al. also used the same waist adjusted weight index to study risk of cognitive decline in Chinese hypertensive patients.

Over the years, alternative obesity indices have been developed in various populations. Okeahialam et al. (6) developed an appliance which measures a novel anthropometric index called Abdominal height which was later tried with huge success in an African population (7). Given differing suitability in different zones, Hung et al. studied multiple alternative indices to detect ranges, temporal trends and their determinants in a Chinese population. As a result, a barrage of data was generated to be deployed in controlling and preventing metabesity related cardiovascular diseases.

A new bent to measures of adiposity and disease outside the cardiovascular system was introduced by Wang et al. They interrogated a link with overactive bladder. Lipid accumulation products and visceral adiposity index were found to predict overactive bladder. These relations had been thought to be inconsistent, though more predictive in females than males as posited in the work of Zhang et al. (8).

In this volume also, Said et al. in trying to push the chronic inflammation basis of obesity related cardiovascular diseases looked at obese children from the perspectives of BMI and IR. Using markers of inflammation and IR, they showed that it was the trajectory obesity took in causing cardiovascular diseases. Obesity associated inflammation has been shown to underpin much of ASCVD as shown by Karakasis et al. (9)

To round up and make a case for regional peculiarities, Vera-Ponce et al. used ATP III and IDF criteria to assess abdominal obesity, and came up with substantial variations. This speaks to the heterogeneity in most of the traditional obesity metrics. This may call for a shift to blood biomarkers for universality.
